# Polarized cellular patterns of endocannabinoid production and detection shape cannabinoid signaling in neurons

**DOI:** 10.3389/fncel.2014.00426

**Published:** 2015-01-06

**Authors:** Delphine Ladarre, Alexandre B. Roland, Stefan Biedzinski, Ana Ricobaraza, Zsolt Lenkei

**Affiliations:** ^1^Brain Plasticity Unit, ESPCI-ParisTechParis, France; ^2^Centre National de la Recherche Scientifique UMR 8249Paris, France

**Keywords:** CB1, DAGL, endocannabinoid, cyclic nucleotide, allosteric, biased agonism, lipid, FRET

## Abstract

Neurons display important differences in plasma membrane composition between somatodendritic and axonal compartments, potentially leading to currently unexplored consequences in G-protein-coupled-receptor signaling. Here, by using highly-resolved biosensor imaging to measure local changes in basal levels of key signaling components, we explored features of type-1 cannabinoid receptor (CB1R) signaling in individual axons and dendrites of cultured rat hippocampal neurons. Activation of endogenous CB1Rs led to rapid, G_i/o_-protein- and cAMP-mediated decrease of cyclic-AMP-dependent protein kinase (PKA) activity in the somatodendritic compartment. In axons, PKA inhibition was significantly stronger, in line with axonally-polarized distribution of CB1Rs. Conversely, inverse agonist AM281 produced marked rapid increase of basal PKA activation in somata and dendrites, but not in axons, removing constitutive activation of CB1Rs generated by local production of the endocannabinoid 2-arachidonoylglycerol (2-AG). Interestingly, somatodendritic 2-AG levels differently modified signaling responses to CB1R activation by Δ^9^-THC, the psychoactive compound of marijuana, and by the synthetic cannabinoids WIN55,212-2 and CP55,940. These highly contrasted differences in sub-neuronal signaling responses warrant caution in extrapolating pharmacological profiles, which are typically obtained in non-polarized cells, to predict *in vivo* responses of axonal (i.e., presynaptic) GPCRs. Therefore, our results suggest that enhanced comprehension of GPCR signaling constraints imposed by neuronal cell biology may improve the understanding of neuropharmacological action.

## Introduction

Polarized neuronal architecture maintains the directionality of information flow through neuronal networks. Accordingly, protein and lipid composition of the plasma membrane greatly differs between axons and the somatodendritic compartment (Horton and Ehlers, 2003). Local interaction between cell membrane components is increasingly considered as a key dynamic component in sensory and signaling pathways. Notably, the highly regulated lipid environment may control the structure, conformation and function of embedded proteins (Phillips et al., 2009). A major brain G-protein coupled receptor (GPCR) that may be particularly sensitive to the lipid composition of the plasma membrane is the type-1 cannabinoid receptor (CB1R). Predominantly localized in axons and specific presynaptic nerve terminals, CB1R is the neuronal target of endocannabinoid lipids (eCBs) and of Δ^9^-tetrahydrocannabinol (THC), the major psychoactive substance of marijuana. CB1Rs may show elevated tonic (constitutive) activation in neurons (Pertwee, 2005), potentially resulting from a combined effect of conformational instability (D'Antona et al., 2006) and ubiquitously present membrane-borne eCBs, such as 2-arachidonoylglycerol (2-AG), which is the most prominent brain eCB (Alger and Kim, 2011; Howlett et al., 2011) as well as an important intermediate in the production of several other bioactive lipids (Nomura et al., 2011). 2-AG is released from cell membrane phospholipids by the action of phospholipase C and diacylglycerol lipases (DAGLα and DAGLβ). eCBs are generally considered to be retrograde signals, being produced in the postsynaptic cell and traveling “backwards” across the synaptic cleft to activate CB1Rs on presynaptic nerve terminals (Freund et al., 2003; Kano et al., 2009). However, in addition to this retrograde synaptic signaling effect, eCBs synthetized in the somatodendritic membrane may also have cell-autonomous effects on local CB1Rs, such as endocannabinoid-mediated somatodendritic slow self-inhibition (SSI) (Bacci et al., 2004; Marinelli et al., 2009) or somatodendritic endocytosis-driven transcytotic targeting (Leterrier et al., 2006; Simon et al., 2013). These findings suggest that locally produced 2-AG may activate somatodendritic CB1Rs, although such CB1R-induced somatodendritic signaling has not yet been shown directly.

CB1R activation, through coupling to G_i/o_ heterotrimeric proteins, leads to inhibition of cyclic adenosine monophosphate (cAMP) production and inhibition of cyclic-AMP-dependent protein kinase (PKA) activity (Howlett, 2005). cAMP and PKA regulate essential biological functions in neurons such as excitability, efficacy of synaptic transmission and axonal growth/pathfinding. Therefore, CB1R coupling to this major signaling pathway may have important consequences on neuronal function. However, in absence of direct measurement of somatodendritic and axonal CB1R signaling, whether and how differences in local CB1R density and local 2-AG content regulate signaling responses to cannabinoids remain unknown.

More generally, it is currently not known how the highly-polarized neuronal membrane environment may shape GPCR signaling. This information may be important to better understand neuronal effects of therapeutic or abused drugs. Indeed, pharmacological response profiles are usually established in non-polarized heterologous expression systems, such as immortalized cell lines, but results derived from these experimental setups may not precisely indicate the pharmacological response that the studied ligand will elicit in polarized neuronal environments, for instance in the extremely thin axons. Therefore, here we used a highly-resolved and sensitive Förster Resonance Energy Transfer (FRET) approach to measure *in vitro* ligand-induced modulation of basal cAMP/PKA levels downstream of endogenous CB1Rs, in individual axons, dendrites, and somata of well-differentiated hippocampal neurons.

## Materials and methods

### Animals

All experiments were performed in agreement with the European Community Council Directive of 22nd September 2010 (010/63/UE) and the local ethics committee (*Comité d'éthique en matière d'expérimentation animale n°59, C2EA – 59, ‘Paris Centre et Sud’*) were used for dissociated cell culture experiments.

### Chemicals, antibodies and DNA constructs

CB1R agonists WIN55,212,2 (WIN), CP55,940 (CP) and 2-arachydonoylglycerol (2-AG), CB1R inverse agonist AM281 (AM) and DAGL inhibitor RHC80267 (RHC) were obtained from R&D Systems Europe. Dimethyl Sulfoxide (DMSO), Tetrahydrolipstatin (THL), Δ^9^-Tetrahydrocannabinol solution (THC), Pertussis Toxin (PTX), Forskolin (Fsk), monoclonal mouse anti-Tau antibody, monoclonal mouse anti-microtubule-associated protein 2 (anti-MAP2) antibody, Bovine Serum Albumin (BSA) and Poly-D-Lysine were obtained from SIGMA-ALDRICH. Polyclonal anti-DAGLα antibody was obtained from Frontier Institute co., ltd (JAPAN). B27, Lipofectamine 2000 and Neurobasal were obtained from Life Technologies.

AKAR4, Lyn-AKAR4 and AKAR4-Kras probes were provided by Dr. Jin Zhang's laboratory (Baltimore, USA). ^T^Epac^VV^ probe provided by Dr. Kees Jalink laboratory (Amsterdam, Netherlands).

### Hippocampal neuronal cultures

Hippocampal neuronal cultures were performed essentially as described previously (Leterrier et al., 2006). Briefly, hippocampi of Sprague–Dawley rat (Janvier) embryos were dissected at embryonic day 18. After trypsinization, dissociation was achieved with a fire-polished Pasteur pipette. Cells were counted and plated on poly-D-lysine-coated 18-mm diameter glass coverslips, at a density of 300–400 cells/mm^2^. The plating medium was Neurobasal supplemented with 2% B27 and containing Stabilized Glutamine (0.5 mM) and penicillin G (10 U/ml)/streptomycin (10 g/ml). Four hours after plating, the coverslips were transferred into Petri dishes containing supplemented Neurobasal medium that had been conditioned for 24 h on a 80% confluent glia layer. Neurons were transfected after 6 days *in vitro* (DIV6) using Lipofectamine 2000, following the manufacturer's instructions.

### FRET imaging

Neurons transfected either with ^T^Epac^VV^ or AKAR4-Kras probes were imaged by videomicroscopy between DIV7 and DIV11 on a motorized Nikon Eclipse Ti-E/B inverted microscope with the Perfect Focus System (PFS) in a 37°C thermostated chamber, using an oil immersion CFI Plan APO VC 60X, NA 1.4 objective (Nikon).

Acquisitions were carried out at the excitation wavelength of the CFP (434 ± 15 nm) using an Intensilight (Nikon). Emitted light passed through an Optosplit II beam-splitter (Cairn Research) equipped with a FF509-FDi01 dichroïc mirror, a FF01-483/32-25 CFP filter and a FF01-542/27-25 YFP filter and was collected by an EM-CCD camera (Evolve 512, Photometrics), mounted behind a 2× magnification lens. Acquisitions were performed by piloting the set-up with Metamorph 7.7 (Molecular Devices). All filter sets were purchased from Semrock.

Cultured neurons on 18-mm coverslips were placed in a closed imaging chamber containing an imaging medium: 120 mM NaCl, 3 mM KCl, 10 mM HEPES, 2 mM CaCl_2_, 2 mM MgCl_2_, 10 mM D-glucose, 2% B27, 0.001% BSA.

We have previously characterized axons and dendrites in our cultures by using immunolabeling for Tau and MAP2 proteins, respectively, that allowed to establish the characteristic morphology of these neurites in cultured hippocampal neurons. Here we have used this morphological criteria to identify axons and dendrites. The acquisition lasted 90 min, recording one image each 2 min, by imaging in parallel 25–30 [10 à15 neurones mais pour chaque neurone: 1 champs sur soma, 1 champ sur l'axone et une champ sur dendrites distales (facultatif)] fields-of view on the same coverslip. 30 min after the beginning of the acquisition, pharmacological treatment was applied then 60 min after the beginning of the acquisition, Forskolin 10 μM was applied.

### FRET data analysis

All imaged neurons were analyzed and included in the final result, except the neurons that matched at least one of the three pre-defined exclusion criteria: (1) lack of response to the terminal Fsk stimulation, (2) loss of focus during the time-lapse sequence, or (3) the impossibility to realign artifactual lateral movement. All key analysis results were obtained by an experimenter blind to the treatment condition.

Images were divided in two parts in ImageJ to separate the CFP channel from the YFP channel. Stacks were realigned to correct for artifactual lateral movement. Data were then analyzed on Matlab by calculating the FRET ratio at each time point for one or several Regions Of Interest (ROIs). The user defined ROIs for each position. For each image, the value of the FRET ratio corresponds to $\frac{IC-BC}{IY-BY}$ for ^T^Epac^VV^ probe and to $\frac{IY-BY}{IC-BC}$ for AKAR4-Kras probe

IY: Mean Intensity of ROI in YFP channel;

BY: Mean Intensity of the background in YFP channel;

IC: Mean Intensity of ROI in CFP channel;

BC: Mean intensity of the background in CFP channel

For each ROI, the FRET ratio was then normalized by the baseline mean, defined as the 7 time points before first treatment application.

FRET Ratio normalized to baseline= 100 ∗ $\frac{Rc-Ro}{Ro}$

Rc: Value of raw FRET ratio

Ro: Mean of the baseline

The quantitative results obtained for each neuronal compartment were grouped together and, for each time point, the mean FRET ratio normalized to baseline and S.E.M. were calculated. Due to CFP photobleaching, FRET ratio tends to increase slowly during the acquisition. This deviation was corrected for somata and dendrites on Matlab. Mean slope was calculated for all neurons in somata and dendrites, respectively, for the last 7 time points before addition of treatment and substracted from all FRET ratio time points. In the axon, precise execution of this correction is not possible. Indeed, as the signal-to-noise ratio is lower in the extremely thin axons as compared to somata and dendrites, the bleaching is “hidden” in the noise, hindering the precise establishment of the correction slope. Thus, we did not correct for CFP photobleaching in the axon.

### FRET statistical analysis

FRET Response was obtained by calculating the mean FRET ratio in Matlab for 6 time points after treatment, from +4 to +14 min (Response).

Groups were compared using GraphPad Prism. Significance of differences between various conditions was calculated using unpaired *t*-tests or one-way ANOVA with Newman-Keuls post-tests for computing p estimates. NS *p* > 0.05, ^*^p < 0.05, ^**^p < 0.01 and ^***^p < 0.001.

### Immunocytochemistry

DIV9 hippocampal cultured neurons were briefly rinsed with Dulbecco's PBS (DPBS; PAA laboratories) and fixed in DPBS containing 4% paraformaldehyde and 4% sucrose. After permeabilization with a 5 min incubation in DPBS containing 0.1% Triton X-100 and blocking for 30 min in antibody buffer (DPBS supplemented with 2% BSA and 3% normal goat serum), neurons were incubated with primary antibodies diluted 1:200 (DAGLα) or 1:250 (MAP2 and Tau) in antibody buffer for 1 h at room temperature. After DPBS rinses, neurons were labeled with secondary antibodies 1:400 in antibody buffer for 30 min at room temperature. Coverslips were fixed with Mowiol containing Hoechst. Images were obtained using a dry 40× objective lens on Zeiss Axio Imager M1. Excitation wavelengths of 488 nm (DAGLα) and 568 nm (MAP2 or Tau) were used.

### Confocal microscopy

Hippocampal cultured neurons were cotransfected at DIV6 with DsRed2 and various FRET probes (^T^Epac^VV^, AKAR4, AKAR4-Kras, Lyn-AKAR4) and fixed in DPBS containing 4% paraformaldehyde and 4% sucrose at DIV7. Images were obtained using an oil immersion objective lens (Plan-Apochromat 60X, NA 1.4) on a Nikon A1 confocal microscope. Excitation wavelengths of 488 nm (FRET probes) and 568 nm (DsRed2) were used. Stacks were obtained with one image per optical section and 300 nm between each section.

## Results

### Endogenous CB1Rs modulate basal PKA activation levels in neurons

**Figure 1:**
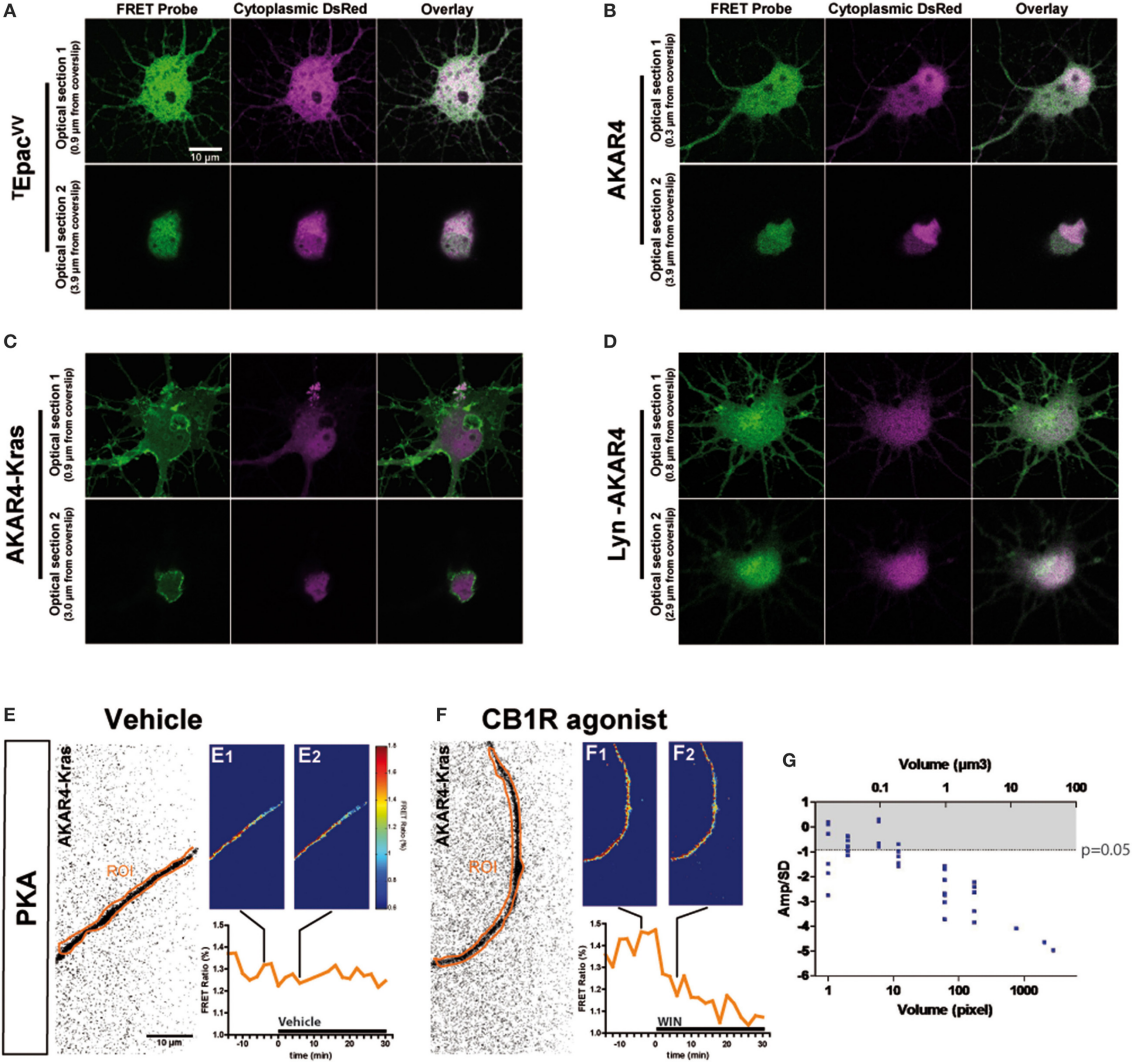
**Quantitative measure of basal cAMP/PKA pathway modulation downstream of endogenous neuronal CB1Rs in small cytoplasmic volumes. (A–D)** Cultured hippocampal neurons expressing soluble (cytoplasmic) DsRed2 and various FRET probes designed to measure cAMP concentration or PKA activity: ^T^Epac^VV^ **(A)**, AKAR4 **(B)**, AKAR4-Kras **(C)**, LYN-AKAR4 **(D)**. After fixation, confocal imaging at two different optical sections shows sub-cellular localization of the probes. AKAR4-Kras probes are well-localized to the plasma membrane in the somatodendritic region. **(E,F)** Modulation of basal PKA activity downstream of endogenous CB1Rs in axonal Regions of Interest (ROI) in AKAR4-Kras expressing neurons. The first image of the acquisition on YFP channel, inverted and with enhanced contrast for better visibility, is shown with the ROI (orange) **(E,F)**. The mean FRET ratio is shown at 4 min (-t4) before **(E_1_,F_1_)** and at 6 min (t6) after **(E_2_,F_2_)** the addition of treatment at t0. Incubation with vehicle does not change the FRET ratio **(E_2_)** compared to baseline **(E_1_)** but addition of agonist WIN 55-212,2 (WIN) 100 nM induces a rapid FRET-ratio decrease **(F_2_)**, as compared to baseline **(F_1_)**. **(G)** Test of FRET imaging sensitivity by determining the smallest axonal cytoplasmic volume allowing the measurement of significant PKA activity decrease after WIN-induced activation of endogenous CB1Rs. We calculated the mean value of the FRET response amplitude normalized to baseline (Amp) and its standard deviation (*SD*), in different axonal ROIs, between t4 (4 min after drug treatment) and t14. The ratio of the FRET response amplitude to its standard deviation (Amp/SD) is represented in function of the volume (see text). The WIN effect is significantly different from control (modeled as an effect of Amp = 0 with the same standard deviation than the corresponding WIN-stimulated response) at the Amp/SD ratio equal to −0.91 (in gray, *p* < 0.05, Student's *t*-test, *N* = 10), which is reached starting from ~1 μm^3^ axonal volume. Data information: Scale bar: 10 μm **(A–F)**.

The characteristic inhibition of cyclic AMP production and PKA activity by G_i/o_-protein coupled GPCRs is usually detected in pharmacological assays after GPCR over-expression and forskolin-induced artificial activation of adenylyl cyclases. Here we aimed to directly measure cannabinoid-induced changes in basal levels of neuronal PKA signaling, downstream of endogenous CB1Rs in cultured hippocampal neurons. Pilot experiments indicated that by using a sensitive EM-CCD camera and hardware-based focus stabilization (see Materials and Methods) we are able to measure cannabinoid-induced inhibition of cyclic AMP production and PKA activity in relatively large cytoplasmic volumes such as neuronal somata by using the soluble ^T^Epac^VV^ probe (Klarenbeek et al., 2011) (Figure 1A) and AKAR4 (Depry et al., 2011) (Figure 1B), respectively. However, smaller diameter neurites such as distal dendrites and axons gave weak (low amplitude) and highly variable responses, leading to a low signal-to-noise ratio, which impeded the reliable measure of the relatively small amplitude cannabinoid-induced changes in the FRET ratio with the AKAR4 probe. To overcome this experimental limitation, we hypothesized that, since the PKA activator cAMP is produced by membrane-bound adenylyl cyclases at the plasma membrane and PKA deactivator phosphodiesterases are cytosolic (Neves et al., 2008), targeting a PKA probe to the plasma membrane may strongly increase experimental sensitivity. Indeed, results of a previous report show both higher FRET responses and higher PKA-sensitive potassium current responses downstream of G_s_-protein activation in dendrites that have a high surface-to-volume ratio as compared to the soma (Castro et al., 2010). Therefore, we expressed separately two membrane-targeted PKA biosensors: AKAR4-Kras (Depry et al., 2011), which is targeted to the non-raft domains of the plasma membrane (Figure 1C), and Lyn-AKAR4 (Depry et al., 2011), which is targeted to the raft regions of the plasma membrane (Figure 1D) in well-differentiated hippocampal neurons. In our experimental conditions, AKAR4-Kras showed a more homogenous distribution that segregated well with the plasma membrane at different optical sections of the somatodendritic domain, while Lyn-AKAR4 was more strongly localized to relatively small membrane microdomains and intracellular structures (Figures 1C,D). In order to focus on plasma-membrane localized endogenous CB1Rs, further experiments were therefore performed using AKAR4-Kras.

Does the membrane-targeted AKAR4-Kras probe permit the measurement of cannabinoid-induced modulation of basal PKA levels downstream of endogenous CB1Rs in all neuronal sub-compartments? We tested the sensitivity of our experimental setup by determining the minimal amount of cytoplasmic volume necessary to the detection of cannabinoid-induced modulation of basal PKA levels, in individual thin (mean diameter = 0.7 μm, see **Figure 3C**) and mature (day *in vitro* 9—DIV9) axons of AKAR4-Kras expressing neurons. By using a large region of interest (ROI) to measure the FRET ratio change, treatment with the synthetic CB1R agonist, WIN55,212-2 (WIN) at 100 nM (Figure 1F), but not with vehicle (Figure 1E), induced a important change of the FRET ratio within 2 min, indicating that CB1R activation induces a decrease of basal PKA activity downstream of endogenous CB1Rs. Measuring the FRET responses in a single axon by gradually decreasing the size of the ROI, we determined the minimum cytoplasmic volume necessary for reliable measurement of the WIN-induced FRET signal change. ROI volumes have been determined as described in “Supplementary Materials and Methods.” We found that a significant decrease of WIN-induced basal PKA activity downstream of endogenous CB1Rs could be measured in volumes as small as 1 μm^3^, which corresponds to 1 femtoliter of axonal cytoplasm (Figure 1G), by using a membrane targeted biosensor, such as AKAR4-Kras, possibly because of the high surface-to-volume ratio of extremely thin neurites.

In conclusion, this experimental approach enables the measurement of modulation of basal neuronal PKA activation levels, downstream of an endogenous G_i/o_ protein coupled receptor, in extremely small cellular volumes, such as the cytoplasm of mature axons.

### Transient somatodendritic CB1Rs constitutively inhibit the cAMP/PKA pathway

**Figure 2:**
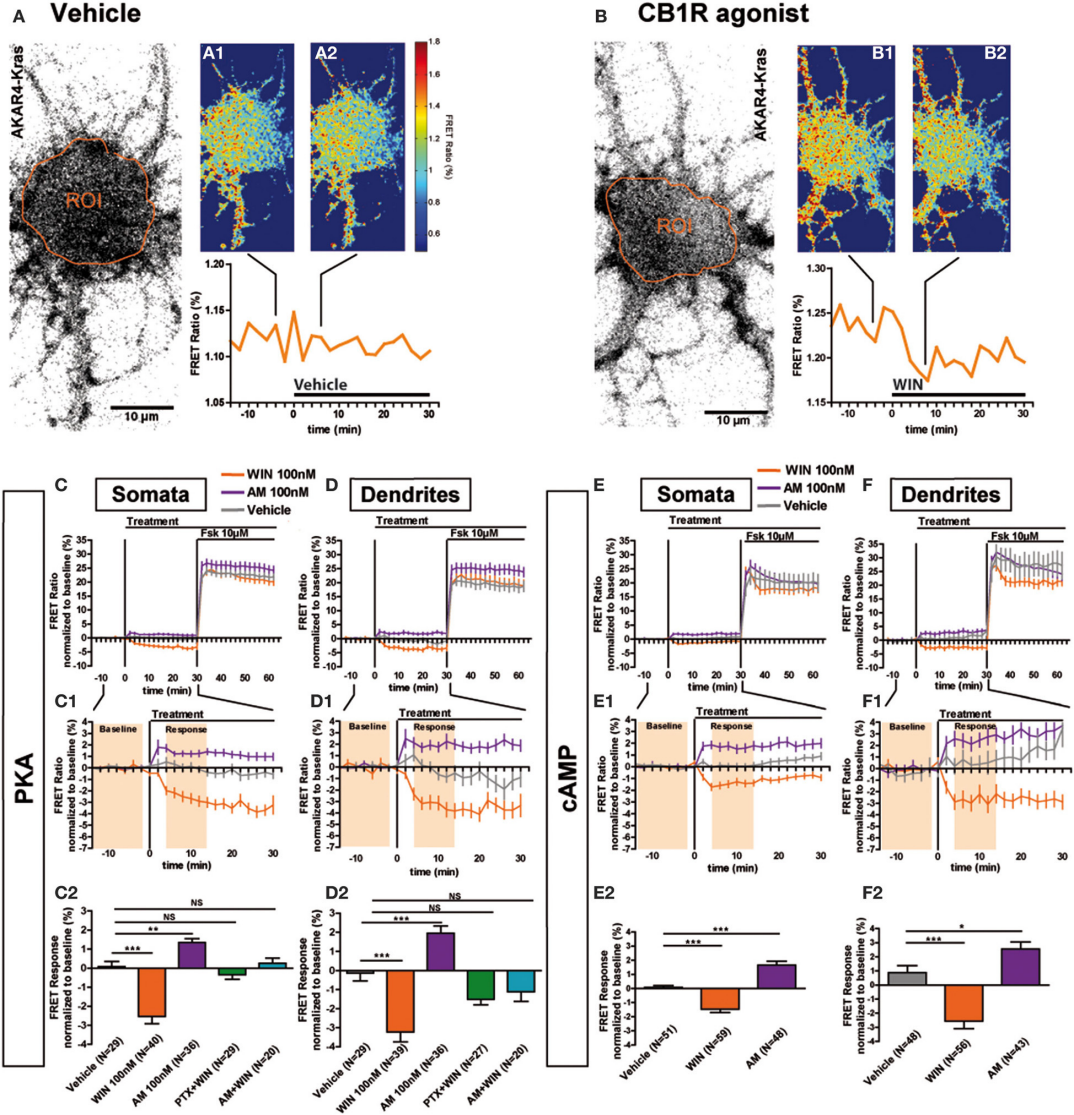
**Somatodendritic CB1Rs constitutively inhibit the cAMP/PKA pathway. (A,B)** Two representative neurons expressing the membrane-targeted PKA sensor AKAR4-Kras. The first image of the acquisition on YFP channel with the ROI is shown **(A,B)**. The mean FRET ratio in somatic ROIs (orange) is shown at 4 min (-t4) before **(A_1_, B_1_)** and at 6 min (t6) after the addition of treatment at t0: Vehicle **(A_2_)** or agonist WIN55-212,2 (WIN) 100 nM **(B_2_)**. **(C–F)** Averaged responses of AKAR4-Kras **(C,D)** or the cAMP sensor ^T^Epac^VV^ expressing neurons **(E,F)**. The FRET ratio normalized to baseline was calculated for each neuron with a time-resolution of 2 min, separately in somata and dendrites. The curves represent mean ± S.E.M. of the FRET ratio for all imaged neurons at each time point. Addition of agonist WIN 100 nM but not of vehicle at t0 results in rapid FRET ratio decrease while inverse-agonist AM281 100 nM (AM) treatment results in increased PKA-activation. At 30 min, adenylyl-cyclase activator Forskolin (Fsk) was added at 10 μM, inducing a saturating increase of the FRET ratio. **C_1_,D_1_,E_1_,F_1_:** Zoom between -t14 and t30 of **C,D,E,F**, respectively, shows significant modulation of basal PKA activity after activation or blockade of CB1Rs. **C_2_,D_2_,E_2_,F_2_:** FRET responses were calculated as the mean response between t4 and t14 min (shaded zone labeled “Response” on **C1**,**D_1_,E_1_,F_1_**), using data normalized to the baseline (shaded zone between -t14 and -t2, labeled “Baseline” on **C_1_**,**D_1_,E_1_,F_1_**), as described in the Materials and Methods Section. Implication of G_i/o_-proteins was shown by the specific inhibitor pertussis toxin (PTX), applied overnight at 100 ng/mL before the beginning of the experiment. The WIN effect was CB1R-induced as shown by pre-treatment with the CB1R-specific antagonist AM281 (1 μM 3 h before the beginning of the experiment). Data information: Data are expressed as mean ± S.E.M.; Statistical analysis was realized with one-way ANOVA followed by Newmann-Keuls post-test; NS *p* > 0.05, ^*^p < 0.05, ^**^p < 0.01, ^***^p < 0.001. Scale bar: 10 μm **(A,B)**.

Previous ultrastructural analysis of hippocampal neurons has shown that in the somatodendritic region, the steady-state presence of endogenous CB1Rs at the plasma membrane is very low both *in vitro* (Leterrier et al., 2006) and *in vivo* (Katona et al., 1999; Thibault et al., 2013). However, previous studies have also reported that most axonally targeted CB1Rs accomplish a transient passage on the somatodendritic plasma membrane (Leterrier et al., 2006; McDonald et al., 2007; Simon et al., 2013). Currently, it remains unknown whether somatodendritic CB1Rs are able to inhibit cAMP/PKA signaling in this neuronal compartment. Therefore, we measured modulation of basal somatodendritic PKA activity downstream of endogenous CB1Rs and found that treatment with WIN at 100 nM, but not with vehicle, induced a moderate decrease of the FRET ratio in individual neurons within a few minutes (Figures 2A,B). To precisely analyze this WIN-induced response, we compared PKA activity in two groups of neurons treated either with vehicle or WIN (100 nM) during 30 min, followed by treatment with the adenylyl cyclase activator Forskolin (Fsk, 10 μM) (Figures 2C,D), to induce a saturating level of AKAR phosporylation, as reported previously (Gervasi et al., 2007). Fsk induced strong somatodendritic PKA activation with a raw baseline-normalized FRET-ratio increase between 20 and 30%. This increase is in the expected range, since activation of AKAR4-Kras in HEK293 cells by addition of 50 μM Fsk induced an increase of 8% of the raw FRET Ratio (Depry et al., 2011). Conversely, activation of CB1Rs with WIN induced a rapid decrease of basal PKA activity in somata (−2.5 ± 0.4%) and dendrites (−3.2 ± 0.5%), which was significant as compared to vehicle (somata: 0.1 ± 0.3%, dendrites: −0.2 ± 0.4%) (Figures 2C_1_,C_2_,D_1_,D_2_). Please note that the measured 2–4% changes of the raw baseline-normalized FRET ratio correspond to 10–20% of the maximal response, which equals typically to 20–25% elevation of the raw baseline-normalized FRET ratio, as established by the terminal saturating Fsk treatment. Given that endogenous CB1Rs are not the only Gα_i/o_-coupled GPCRs in hippocampal neurons, mobilization of the cAMP/PKA pathway in the 10–20% range of the maximal response suggests physiological relevance. Moreover, FRET responses showed Gaussian distribution pattern (as verified by the normality test), indicating that hippocampal neurons did not segregate into sub-populations regarding the effects of CB1R agonist/antagonist application (Supplementary Figures 1A–C). This is in contrast to a previous *ex-vivo* report that studied somatic slow self-inhibition in cortical neurons, where only a subpopulation of neurons was responsive to cannabinoid treatment (Marinelli et al., 2009). The effect of WIN was blocked after overnight treatment with 100 ng/mL of the G_i/o_-protein specific inhibitor pertussis toxin (PTX) (somata: −0.3 ± 0.2%, dendrites: −1.5 ± 0.3%) as well as after 3 h pre-treatment with 1 μM of the CB1R-specific antagonist/inverse-agonist AM281 (somata: 0.3 ± 0.3%, dendrites: −1.1 ± 0.5%). Therefore, endogenous CB1Rs, transiently present on the somatodendritic plasma membrane, can be activated by exogenous cannabinoids and are able to subsequently inhibit basal PKA signaling through their coupling to G_i/o_ proteins both in somata and dendrites.

We have previously reported that somatodendritic CB1Rs are constitutively endocytosed because of constitutive receptor activation, which can be inhibited by pharmacological or genetic tools (Leterrier et al., 2006; Simon et al., 2013). To investigate whether CB1Rs also constitutively inhibit cAMP/PKA signaling in the somatodendritic compartment, we applied the CB1R inverse agonist, AM281 at 100 nM, to neurons expressing AKAR4-Kras. This treatment led to a rapid and significant increase of the FRET ratio both in somata and dendrites (somata: 1.3 ± 0.2%, dendrites: 2.0 ± 0.4%) (Figures 2C_1_,C_2_,D_1_,D_2_). Therefore, somatodendritic CB1Rs exert a constitutive inhibition on PKA activity that is removed by inverse agonist treatment.

Taken together these results indicate that somatodendritic CB1Rs constitutively inhibit PKA activity through the mobilization of G_i/o_ proteins, which is likely due to the inhibition of adenylate cyclase and subsequent decrease of cAMP production. To confirm this mechanism, we directly measured the modulation of basal somatodendritic cAMP concentration, downstream of CB1Rs, by using the ^T^Epac^VV^ probe (Klarenbeek et al., 2011). The activation of endogenous CB1Rs with WIN (100 nM) induced a rapid and significant decrease of cAMP concentration in somata (−1.5 ± 0.3%) and dendrites (−2.6 ± 0.6%), while application of the inverse agonist AM281 at 100 nM led to a rapid and significant increase of cAMP concentration both in somata (1.7 ± 0.3%) and dendrites (2.6 ± 0.5%) (Figures 2E,E_1_,E_2_,F,F_1_,F_2_). Responses to the final 10 μM Fsk treatment are also a slightly different. However, accurate measure of ligand-induced modifications of artificial adenylyl cyclase stimulation by Fsk was not the scope of the present study, where we focused on endogenous CB1R-induced modification of basal PKA activation levels.

These results show that endogenous CB1Rs exert a constitutive inhibition on the cAMP/PKA signaling pathway both in somata and dendrites. In addition, somatodendritic CB1Rs can be further activated by exogenous cannabinoids leading to a rapid decrease of cAMP concentration and PKA activity through activation of G_i/o_ proteins.

### Axonal CB1R signaling is different from dendritic signaling

**Figure 3:**
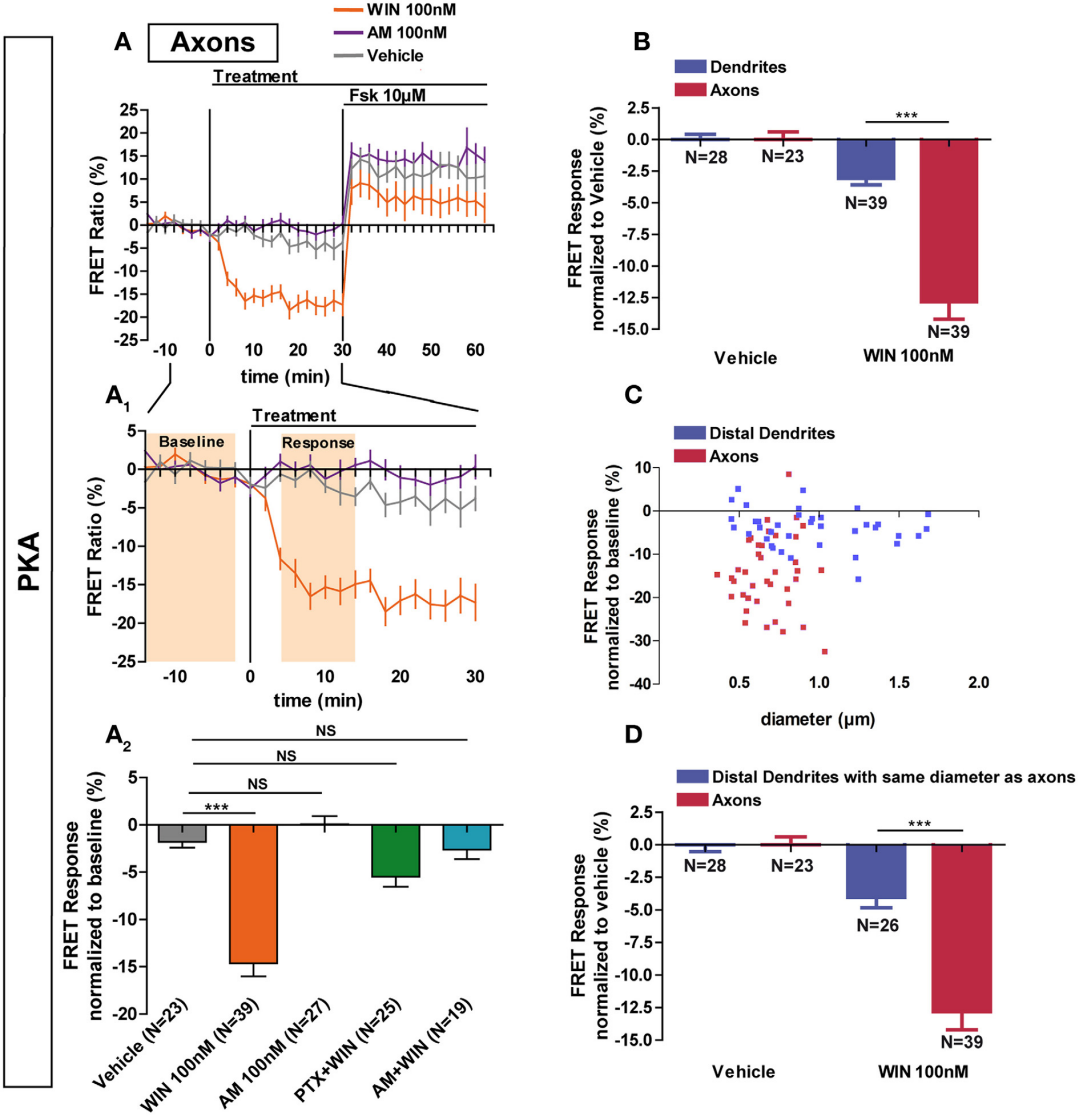
**Axonal CB1R signaling differs from dendritic signaling. (A)** Averaged axonal responses of AKAR4-Kras expressing neurons, as shown on Figures 1E,F. The FRET ratio normalized to baseline was calculated for each neuron with a time-resolution of 2 min. The curves represent mean ± S.E.M. of the FRET ratio for all imaged neurons at each time point. Addition of agonist WIN 100 nM but not of vehicle or inverse-agonist AM281 100 nM (AM) at t0 results in rapid high-amplitude FRET ratio decrease. At 30 min, adenylyl-cyclase activator Forskolin (Fsk) was added at 10 μM, inducing a saturating increase of the FRET ratio. **A_1_:** Zoom between −t14 and t30 of **A** shows significant modulation of basal PKA activity after activation of CB1Rs. **A2:** FRET responses were calculated as the mean response between t4 and t14 min (shaded zone labeled “Response” on **A_1_**), using data normalized to the baseline (shaded zone between −t14 and −t2, labeled “Baseline” on **A_1_**). Implication of G_i/o_-proteins was shown by the specific inhibitor pertussis toxin (PTX), applied overnight at 100 ng/mL before the beginning of the experiment. The WIN effect was CB1R-induced as shown by pre-treatment with the CB1R-specific antagonist AM281 (1 μM, 3 h before the beginning of the experiment). **(B)** Vehicle-normalized FRET response to WIN is significantly stronger in axons than in dendrites. **(C)** Individual FRET responses in axons and distal dendrites are represented in function of their respective diameter. For each group (distal dendrites and axons), a Pearson correlation test was calculated showing no correlation between FRET response and diameter (*r*_distal dendrites_ = −0.065 and *r*_axons_ = 0.03649). **(D)** Distal dendrites having the similar diameter than axons still display significantly weaker vehicle-normalized FRET responses to WIN compared to axons. Data information: Data are expressed as means ± S.E.M.; Statistical analysis was realized with one-way ANOVA followed by Newmann-Keuls post-test **(A_2_)** or unpaired *t*-test **(B,D)**; NS *p* > 0.05, ^***^p < 0.001.

Previous studies have found a polarized accumulation of transcytosed CB1Rs on the axonal plasma membrane due to reduced internalization levels as compared to dendrites (Leterrier et al., 2006; McDonald et al., 2007; Simon et al., 2013). We asked whether the recruitment of signaling pathways downstream of CB1Rs in axons also differ from somata and dendrites. Application of 100 nM WIN led to a rapid and significant decrease of basal PKA activity in axons (−14.6 ± 1.4%) compared to vehicle (−1.8 ± 0.6%) (Figures 3A,A_1_,A_2_). This effect was blocked by pre-treatment with AM281 1 μM (−2.6 ± 1.0%) and PTX 100 ng/mL (−5.5 ± 1.1%), showing that PKA inhibition is specifically mediated by CB1Rs acting through G_i/o_ proteins. In addition, CB1R activation decreased PKA activity more strongly in the axon than in dendrites (dendrite response normalized to vehicle: −3.1 ± 0.5%, axonal response normalized to vehicle: −12.8 ± 1.4) (Figure 3B). Interestingly, in contrast to dendrites, application of the inverse agonist AM281 at 100 nM did not induce a detectable change of PKA activity in the axon (0.1 ± 0.8%), suggesting that axonal CB1Rs are not constitutively activated (Figures 3A,A_1_,A_2_).

Why does axonal CB1R activation lead to a significantly higher amplitude of PKA inhibition in axons than in dendrites and somata? First, similarly to their distribution *in vivo* (Katona et al., 2001; Bodor et al., 2005; Thibault et al., 2013), CB1Rs display an axonally polarized distribution in cultured neurons (Coutts et al., 2001; Leterrier et al., 2006; McDonald et al., 2007; Simon et al., 2013). Second, theoretical models predict, and experiments show that, for signaling molecules produced at the plasma membrane and degraded in the cytoplasm, such as cAMP, the ratio of the surface area of the plasma membrane to the cytoplasmic volume [surface/volume ratio (S/V)] becomes important (Neves et al., 2008). As such, we asked whether the strong decrease of PKA activity observed after CB1R activation in the axon is related to the high S/V ratio of this compartment. However, for both axons and distal dendrites, we found no correlation between neurite diameter and FRET response amplitude after CB1R activation (Pearson's correlation coefficient: *r*_distal dendrites_ = −0.065 and *r*_axons_ = 0.03649) (Figure 3C). Moreover, a sub-population of distal dendrites has the same diameter range as axons. In these thin dendritic segments, the amplitude of the FRET response after CB1R activation was again significantly different from the axonal response (dendrites normalized to vehicle: −4.0 ± 0.8%, axons normalized to vehicle: −12.8 ± 1.4) (Figure 3D). Therefore, morphological differences between axons and dendrites do not explain the observed signaling disparity among these two compartments, suggesting that the polarized distribution of neuronal CB1Rs is the main reason for the enhanced agonist response in axons.

### Constitutive activation of somatodendritic CB1Rs requires local synthesis of endocannabinoids

**Figure 4:**
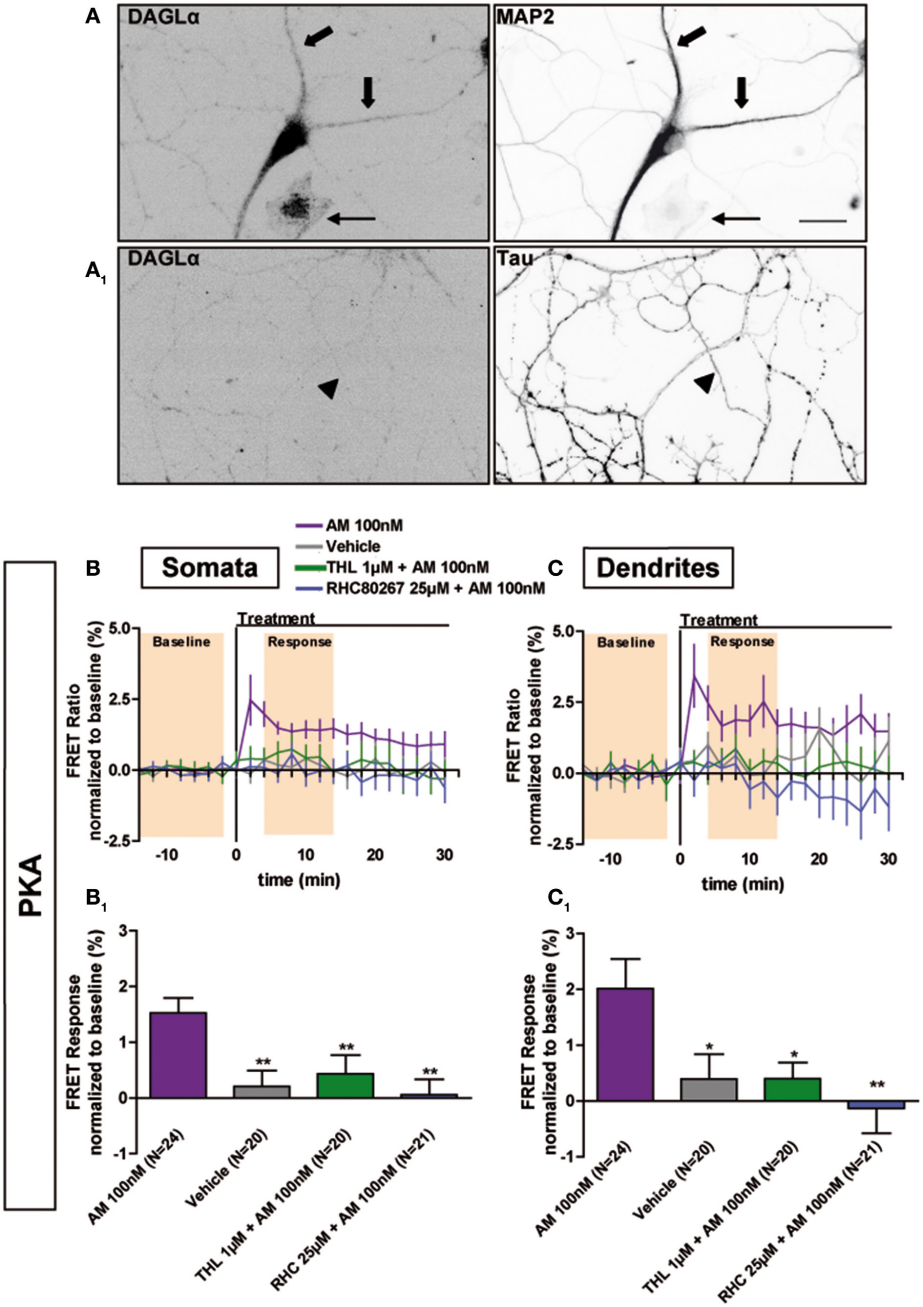
**Constitutive activation of somatodendritic CB1Rs requires locally synthesized endocannabinoids. (A)** Simultaneous immunolabeling of fully-polarized (DIV9) neurons with anti-DAGLα antibody and either anti-MAP2 **(A)** or anti-Tau **(A_1_)** antibodies. Large arrows indicate dendrites, arrow-heads indicate axon and the thin arrow shows an astrocyte. **(B,C)** Averaged somatic and dendritic responses of AKAR4-Kras expressing neurons to inverse-agonist AM281 (AM), with or without inhibiting DAGL activity. The FRET ratio normalized to baseline was calculated for each neuron with a time-resolution of 2 min. The curves represent mean ± S.E.M. of the FRET ratio for all imaged neurons at each time point. Addition of 100 nM AM but not of vehicle at t0 results in elevated PKA activity, revealing constitutive CB1R activation, which is significantly decreased after DAGL inhibition either by tetrahydrolipstatin (THL) 1 μM or RHC80267 25 μM. Data information: Data are expressed as mean ± S.E.M.; Statistical analysis was realized with one-way ANOVA followed by Newmann-Keuls post-test; ^*^p < 0.05, ^**^p < 0.01. Scale bar: 20 μm **(A,A1)**.

Next we investigated why CB1Rs are constitutively activated in the somatodendritic compartment but not in the axon, by focusing on the contribution of endocannabinoids, which play an important role in basal CB1R activation in several experimental systems (Turu et al., 2007; Howlett et al., 2011). The major endocannabinoid 2-arachidonoylglycerol (2-AG) is a lipid molecule present in the cell plasma membrane and is synthesized by DAG Lipases (DAGL). DAGLα, the major DAGL in the postnatal brain, is segregated to axonal tracts during embryonic development but was shown to accumulate after birth in the somatodendritic plasma membrane in several brain areas, such as the cerebellum (Bisogno et al., 2003), striatum (Uchigashima et al., 2007), hippocampus (Katona et al., 2006; Yoshida et al., 2006) and amygdala (Yoshida et al., 2011). Similarly, we found a somatodendritic segregation of DAGLα in fully-polarized (DIV9) cultured hippocampal neurons, while no labeling was found in the axon (Figures 4A,A_1_). This indicates local production of 2-AG in the plasma membrane of the somatodendritic compartment but not in the axonal counterpart. To investigate whether such polarized 2-AG production may explain the aforementioned differences in constitutive CB1R activation between dendrites and axons, we pre-treated neurons expressing the AKAR4-Kras probe with the DAGL inhibitors, Tetrahydrolipstatin (THL) or RHC80267 (RHC), during 3 h before treatment with the inverse agonist AM281 100 nM (Figures 4B,B_1_,C,C_1_). The FRET ratio did not increase in these neurons after adding AM281, neither in somata (AM281: 1.6 ± 0.3%, vehicle: 0.2 ± 0.3%, AM281 after THL 1 μM: 0.4 ± 0.3%, AM281 after RHC 25 μM: 0.1 ± 0.3%) nor in dendrites (AM281: 2.0 ± 0.5%, vehicle: 0.4 ± 0.4%, AM281 after THL 1 μM: 0.4 ± 0.3%, AM281 after RHC 25 μM: −0.1 ± 0.5%). Thus, the constitutive inhibition on PKA activity was removed by DAGL blockade, demonstrating that constitutive activation of somatodendritic CB1Rs requires locally produced 2-AG.

### Signaling responses to exogenous ligands WIN, CP55,940 and Δ^9^-THC are differentially shaped by local production of 2-AG in the somatodendritic compartment

**Figure 5:**
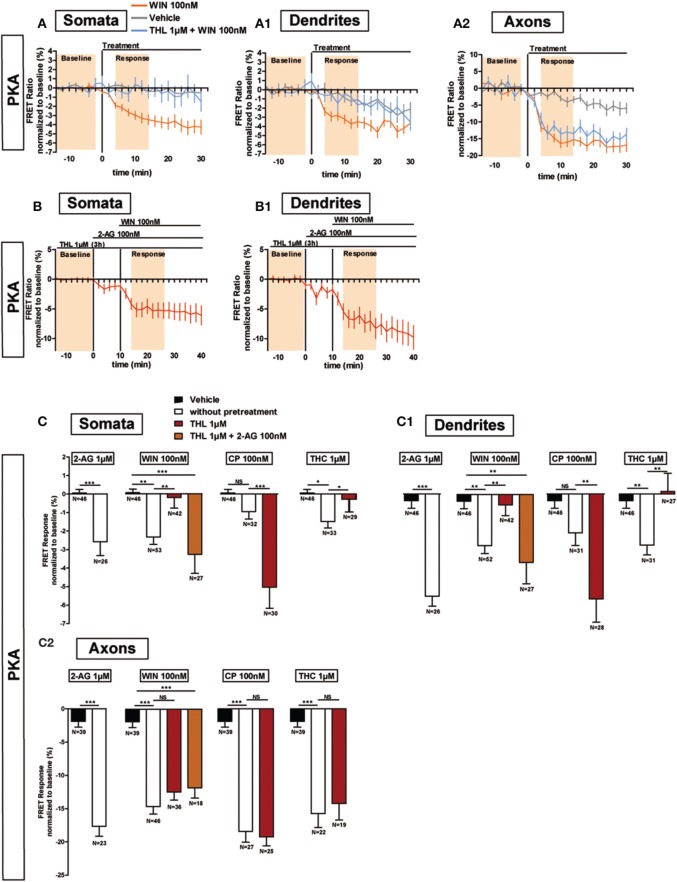
**Endogenous 2-AG significantly modifies CB1R responses to exogenous cannabinoids. (A,B)** Averaged somatic, dendritic and axonal responses of AKAR4-Kras expressing neurons to agonist WIN 55-212,2 (WIN). The FRET ratio normalized to baseline was calculated for each neuron with a time-resolution of 2 min. The curves represent mean ± S.E.M. of the FRET ratio for all imaged neurons at each time point. Addition of WIN 100 nM but not of vehicle at t0 results in decreased PKA activity, which effect is significantly inhibited after DAGL inhibition by tetrahydrolipstatin (THL) 1 μM in somata **(A)** and dendrites **(A_1_)** but not in axons **(A_2_)**. The effect of THL pre-treatment on the WIN effect in somata **(B)** and dendrites **(B1)** can be rescued by applying 2-AG at 100 nM 10 min before WIN. **(C)** Variation of neuronal 2-AG levels (similarly to **A,B**) modifies the FRET responses to exocannabinoids WIN55-212,2 100 nM (WIN), CP55,940 100 nM (CP) and Δ^9^-THC 1 μM (THC), shown as the mean response between t4 and t14 min (shaded zone labeled “Response” on **A,B**), using data normalized to the baseline (shaded zone between −t14 and −t2, labeled “Baseline” on **A,B**) in somata **(C)**, dendrites **(C_1_)** or axons **(C_2_)**. 2-AG levels were reduced by THL 1 μM, applied 3 h before the beginning of the experiment and rescued by 2-AG 100 nM at 10 min before agonist treatment. Data information: Data are expressed as mean ± S.E.M.; Statistical analysis was realized with unpaired *t*-test (2-AG) or one-way ANOVA followed by Newmann-Keuls post-test (WIN, CP, and THC); NS *p* > 0.05, ^*^p < 0.05, ^**^p < 0.01 and ^***^p < 0.001.

Previous results show that, after DAGL inhibition, the amount of CB1Rs increase on the plasma membrane, both in the somatodendritic compartments of neurons and in CHO cells (Turu et al., 2007). In CHO cells, the elevated CB1R levels at the plasma membrane yield enhanced G-protein activation following WIN administration (Turu et al., 2007). We asked whether the THL-induced accumulation of CB1Rs on the somatodendritic membrane is able to produce similar enhanced inhibition of PKA activity after WIN administration, as compared to basal conditions. Therefore, we pre-treated neurons with THL (1 μM) during 3 h before acquisition and applied WIN during the FRET acquisition (Figure 5A). Surprisingly, DAGL inhibition blocked the effect of 100 nM WIN in the somatodendritic compartment instead of signaling enhancement (somatic response to WIN 100nM: −2.4 ± 0.4%, *P* < 0.01 compared to vehicle (0.1 ± 0.2%) and *P* < 0.01 compared to response to WIN 100 nM after 3 h THL 1 μM (−0.2 ± 0.6%), one-way ANOVA followed by Newman-Keuls post-test; dendrite response to WIN 100 nM: −2.8 ± 0.4%, *P* < 0.01 compared to vehicle (−0.4 ± 0.4%) and *P* < 0.01 compared to response to WIN 100 nM after 3 h THL 1 μM (−0.6 ± 0.6%), one-way ANOVA followed by Newman-Keuls post-test) (Figures 5A,A_1_,C,C_1_), while it did not change the FRET response in the axon (response to WIN 100 nM: −14.6 ± 1.1%, *P* < 0.001 compared to vehicle (−1.9 ± 0.9%) and *P* > 0.05 compared to WIN 100 nM after 3 h THL 1 μM (−12.4 ± 1.2%), one-way ANOVA followed by Newman-Keuls post-test) (Figures 5A_2_,C_2_). This suggests that a local 2-AG production drop, caused by THL pre-treatment, was responsible for the somatodendritic signaling decrease, which indeed could be rescued by 2-AG (100 nM), applied for 10 min before WIN treatment (somata: −3.3 ± 1.0%, dendrites: −3.7 ± 1.2%, axons: −11.8 ± 1.6%; WIN responses were compared to vehicle) (Figures 5B,B_1_,C,C_1_,C_2_). By itself, 2-AG used at 1 μM is able to decrease PKA activity in all neuronal compartments, with a stronger effect in axons as compared to the somatodendritic compartment (somata: −2.6 ± 0.7%, dendrites: −5.5 ± 0.5%, axons: −17.7 ± 1.5%) (Figures 5C,C_1_,C_2_). To verify if the presence of local 2-AG is a general requirement for somatodendritic CB1R activation, we tested two other, structurally different, CB1R agonists: CP55,940 (CP) and Δ^9^-THC (THC), the psychoactive compound of marijuana. In control neurons, the effect of CB1R activation with 100 nM CP was comparable to WIN, with a decrease of PKA activity in both dendrites and axons as well as a stronger amplitude in the axonal response compared to dendrites (somata: −1.0 ± 0.4%; dendrites: −2.1 ± 0.7%; axons: −18.4 ± 1.6%) (Figures 5C,C_1_,C_2_). However, blockade of DAGL by THL pretreatment did not decrease the effect of CP (100 nM) in the somatodendritic compartment. On the contrary, and according to our previous expectations for WIN, this response was enhanced as compared to control neurons (somata: −5.0 ± 1.2%, dendrites: −5.7 ± 1.3%, axons: −19.2 ± 1.4%; responses to CP 100 nM after 3 h THL 1 μM were compared to CP 100 nM alone). Finally, treatment with THC (1 μM) also decreased PKA activity in all neuronal compartments, with a stronger effect in the axon compared to the somatodendritic compartment (somata: −1.5 ± 0.4%, dendrites: −2.8 ± 0.5%, axons: −15.8 ± 2.1%) (Figures 5C,C_1_,C_2_). However, the somatodendritic effect of 1 μM THC was suppressed by THL pretreatment while it did not affect the axonal response (somata: −0.3 ± 0.7%, dendrites: 0.1 ± 1.0%, axons: −14.2 ± 2.5%; responses to THC 1 μM after 3 h THL 1 μM were compared to THC 1 μM alone) (Figures 5C,C_1_,C_2_). Thus, THC and WIN require local presence of 2-AG to activate somatodendritic CB1Rs.

In conclusion, activation of CB1Rs by exogenous cannabinoids can have contrasted effects on the mobilization of somatodendritic signaling pathways: these effects are highly shaped by local presence of 2-AG which is necessary for the effects of both WIN and THC, but not of CP, in this neuronal compartment.

## Discussion

We developed a highly sensitive quantitative *in vitro* method to evaluate, for the first time to our knowledge, the modulation of the cAMP/PKA signaling pathway downstream of an endogenous G_i/o_ protein coupled receptor with sub-neuronal resolution. We measured modulation of basal cAMP/PKA signaling, after activation or blockade of endogenous CB1Rs, in somata, dendrites and axons of well-differentiated cultured rat hippocampal neurons. Our results show that polarized distribution of two neuronal proteins, the endocannabinoid synthesizing DAGLα enzyme and the CB1R, leads to previously unappreciated quantitative sub-domain dependent differences in intraneuronal GPCR signaling. In axons, the combined effect of high CB1R density and absence of DAGLα activity leads both to elevated response amplitude following agonist stimulation, as well as to a lack of constitutive activation. In the somatodendritic compartment, relatively low CB1R density and high DAGLα activity, locally producing the membrane component endocannabinoid 2-AG, results in constitutive activation of CB1R-activated signaling which is accompanied by significant but relatively low amplitude agonist-induced signaling responses.

In addition, we show that the 2-AG content of the somatodendritic plasma membrane has contrasted effects on CB1R activation by various exogenous cannabinoid ligands: at the ligand concentrations used in the present study, CP acts as a classical agonist while both WIN and THC require the presence of endogenous 2-AG to efficiently activate CB1Rs.

### CB1Rs constitutively inhibit cAMP/PKA signaling in the somatodendritic compartment but not in the axon

Several studies reported that CB1Rs display constitutive activity in neurons (Pan et al., 1998; Hillard et al., 1999) and notably, these receptors are constitutively endocytosed in the somatodendritic compartment, but not in axons, due to basal activation (Leterrier et al., 2006; Simon et al., 2013). Here we show that application of the inverse agonist AM281 leads to a rapid increase in both somatodendritic cAMP concentration and PKA activity, suggesting constitutive CB1R activation in the somatodendritic compartment but not in the axon. In non-polarized cells, constitutive CB1R activity is highly diminished in the absence of endocannabinoid 2-AG (Turu et al., 2007). We found here that DAGLα is segregated in the somatodendritic compartment and its inhibition removes the effect of AM281. Therefore, somatodendritic CB1Rs are constitutively activated by a high-tone of locally produced 2-AG, and the lack of constitutive activity in the axon is due to the absence of 2-AG.

Our results show important somatodendritic effects on cAMP/PKA regulation for an axonal (i.e., presynaptic) receptor. Previously, CB1R-mediated somatodendritic slow self-inhibition (SSI) was reported in neocortical interneurons (Bacci et al., 2004) and pyramidal neurons (Marinelli et al., 2009). During SSI, activation-induced post-synaptic increase of calcium stimulates somatodendritic DAGL, leading to local 2-AG production and cell-autonomous activation of somatodendritic CB1Rs and G protein inwardly rectifying K^+^ (GIRK) channels (Marinelli et al., 2008). Our results are coherent with these observations and extend the mechanical understanding of the phenomenon. βγ subunits of G_i/o_ proteins may directly activate GIRK channels (Lujan et al., 2009). Here we directly demonstrate that such G_i/o_ proteins can be activated by CB1Rs in the somatodendritic region and we show that this activation impacts on local cAMP and PKA activation levels. Therefore, it is likely that SSI-inducing activation leads to a parallel decrease of somatodendritic cAMP levels and to PKA inhibition. Interestingly, while cortical neurons are segregated into sub-populations that respond differently to CB1R activation *ex vivo* (Marinelli et al., 2009), our results, which show a Gaussian distribution in responses (Supplementary Figure 1), suggest that either such sub-populations are not present in hippocampal neurons or that our technique is not sensitive enough to detect such differences. We also report that basal production of 2-AG is both necessary and sufficient to activate G_i/o_ proteins through CB1Rs to achieve measurable constitutive inhibition of somatodendritic cAMP/PKA signaling. GPCRs may also display constitutive activity due to conformational instability (Kenakin, 2004) and several studies reported that CB1Rs may display constitutive activity in systems apparently free of endocannabinoids (review in Pertwee, 2005). However, it is difficult to formally exclude the presence of endocannabinoids, since these lipid molecules may be present in cell plasma membrane at high levels even in non-stimulated neurons (Alger and Kim, 2011).

Here, our results indicate the complete elimination of measurable constitutive somatodendritic CB1R activation after pharmacological inhibition of DAGL and the lack of constitutive activation in the mature axon, where the absence of DAGL suggests low levels of membrane-borne 2-AG. However, a certain level of conformational instability may be necessary to enable constitutive activation of CB1R by 2-AG. Alanine substitution of the T210 residue, which is located in the 3rd transmembrane helix and is well-conserved in the cannabinoid receptor family but absent in other class A GPCRs (D'Antona et al., 2006), results in change of the CB1R conformational state (Simon et al., 2013) and yields a hypoactive receptor form, which displays significantly lower constitutive activity but preserves responsiveness to agonists (D'Antona et al., 2006). Overexpressed T210A mutant CB1Rs accumulate on the somatodendritic surface because of reduced steady-state endocytosis and this accumulation leads to elevated somatodendritic responses to WIN treatment (Simon et al., 2013). To further understand the effect of conformational instability induced by T210 on CB1R signaling, it would be useful in the future to induce the T210A mutation in the endogenous CB1R through a genetic editing approach, in order to avoid the putative effects of receptor overexpression on the signaling response.

### CB1R activation by exogenous cannabinoids in axons differs from that in dendrites, where local 2-AG modulates the response to agonists

Activation of endogenous CB1Rs leads to a stronger decrease of PKA activity in axons compared to dendrites. This difference is not due to the shape of neurites. CB1Rs are enriched in the axonal plasma membrane, leading to approximately 10-fold more endogenous CB1Rs receptors at the plasma membrane in axons as compared to dendrites (McDonald et al., 2007). Here, we observed that the decrease of PKA activity after CB1R activation is about 3-fold stronger in the axon than in dendrites. Thus, differences in sub-neuronal signaling and receptor density are in the same range, suggesting that the main cause of the polarized signaling response is polarized CB1R distribution. Our previous results have shown that polarized spatial distribution of CB1Rs is precisely regulated by steady-state somatodendritic activation and endocytosis coupled to trans-cytotic targeting (Simon et al., 2013), so it is likely that polarized distribution (i.e., somatodendritic segregation) of DAGLα is the principal cause of the polarized distribution of CB1Rs. However, this model is based on previous data obtained by using a highly-sensitive quantitative experimental approach employing overexpressed epitope-tagged CB1Rs and exogenous cannabinoids (Simon et al., 2013). In future studies, it would be interesting to verify this hypothesis with sensitive detection of endogenous CB1R localization and well-controlled modification of cell-autonomous endocannabinoid levels.

Our results also indicate that inhibition of 2-AG synthesis prevents WIN-induced activation of CB1Rs in the somatodendritic compartment, whereas, in the axon, absence of 2-AG leads to a lack of constitutive activity but does not prevent activation by WIN. Presently, possible interactions between 2-AG and WIN on CB1R activation are not clearly understood. CB1R intramembrane loops were proposed to shape a “binding pocket” that 2-AG could reach through a gap allowing lipidic ligands to enter from membrane bilayer, without need of extracellular access (Hurst et al., 2013). Aminoalkylindole cannabinoids such as WIN bind at a different site (McAllister et al., 2003; Hurst et al., 2013), so WIN could act as a positive allosteric modulator for 2-AG, by increasing 2-AG-induced constitutive CB1R activation, leading to enhanced inhibition of cAMP/PKA signaling in the somatodendritic compartment. Interestingly, the agonist CP55,940 binds at a different site than WIN (Kapur et al., 2007) and dissimilarly to WIN, CP55,940-mediated inhibition of somatodendritic PKA activity is significantly stronger after DAGL inhibition. After DAGL inhibition, CB1R levels increase at the somatodendritic plasma membrane because of reduced endocytic elimination (Turu et al., 2007) possibly explaining the enhanced CP effect in the somatodendritic compartment. In the axon, CP-induced PKA activity decrease is not modified by THL, as DAGL is absent in this compartment. Finally, the phytocannabinoid THC induces a decrease of PKA activity in the somatodendritic compartment that is removed after DAGL inhibition. Therefore, neuronal pharmacology of THC is similar to WIN but not to CP, suggesting that THC may also act as an exogenous positive allosteric modulator, that amplifies the CB1R-activating effect of locally produced 2-AG in the somatodendritic compartment. These surprising interactions between 2-AG and exogenous cannabinoid ligands may result from changes in CB1R levels on the somatodendritic surface but also from different, potentially overlapping and to date not completely understood mechanisms, such as conformation-induced changes in ligand affinity and efficiency and competition for ligand binding sites. Full comprehension of these effects requires further technical development that, through enhancing the sensitivity of the experimental approach presented here, may allow detailed pharmacological characterization in the future, such as precise measurement of ligand affinity and efficacy, of endogenous GPCR signaling in neuronal sub-domains.

In conclusion, our results show that pharmacological responses to activation of a major neuronal GPCR are different in axons and dendrites. In the somatodendritic compartment, CB1Rs are constitutively activated by locally produced 2-AG, constitutively inhibit the cAMP/PKA pathway and can be further activated, significantly albeit moderately, by exogenous cannabinoids. A similar activation profile was reported in non-polarized cells (Turu et al., 2007). However, the pharmacological profile of axonal CB1Rs is different: their activation leads to a strong decrease of PKA activity and no significant constitutive activation is observed. This highly contrasted difference in sub-neuronal signaling responses warrants caution in extrapolating pharmacological profiles, which are typically obtained in non-polarized cells, to predict *in vivo* responses of axonal (i.e., presynaptic) GPCRs. Therefore, the *in situ* pharmacological approach presented in our study may also be useful for a better understanding of the physiology of other neuronal GPCRs.

## Author contributions

Delphine Ladarre and Zsolt Lenkei designed the experiments, Delphine Ladarre, Alexandre B. Roland, Stefan Biedzinski and Ana Ricobaraza performed the experiments, Delphine Ladarre, and Stefan Biedzinski analyzed the data and Delphine Ladarre and Zsolt Lenkei wrote the paper.

### Conflict of interest statement

The authors declare that the research was conducted in the absence of any commercial or financial relationships that could be construed as a potential conflict of interest.
