# Supplementary material for: Polarized cellular patterns of endocannabinoid production and detection shape cannabinoid signaling in neurons
Source: Front Cell Neurosci. 2015 Jan 6;8:426. doi: 10.3389/fncel.2014.00426 (PMC4285097; doi:10.3389/fncel.2014.00426)
Supplement: Supplementary file 1 [file DataSheet1.DOCX]

***Supplementary Material***

**Polarized cellular patterns of endocannabinoid production and detection shape cannabinoid signaling in neurons**

*Ladarre Delphine^1,2^, Roland Alexandre^1,2,#^, Biedzinski Stefan^1,2^, Ricobaraza Ana^1,2^, Lenkei Zsolt^1,2,*^*

*^1^Brain Plasticity Unit, ESPCI-ParisTech, Paris, France*

*^2^CNRS UMR* *8249, Paris, France*

^*^ Corresponce:

Zsolt Lenkei M.D., Ph.D., Brain Plasticity Unit, ESPCI-ParisTech, 10, rue Vauquelin, 75005 Paris, France. zsolt.lenkei@espci.fr

^#^ Current address:

FAS Center for Systems Biology, Harvard University, Cambridge, MA, USA

**Supplementary Material and Methods**

*Pseudocolor images*

On Figures 1E_1_-E_2_, 1F_1_-F_2_, 2A_1_-A_2_ and 2B_1_-B_2_, pseudocolor images are used in order to show FRET ratio changes after treatment addition. The FRET ratio calculated on these figures is not normalized by the baseline. For each pseudocolor image, both images from CFP and YFP channels have been filtered on Image J, using “mean of 2 pixels”. Then, a MatLab algorithm (see below) calculated the ratio YFP/CFP pixel per pixel without background substraction. Finally, a threshold has been manually applied in order that background pixels value equals zero.

Matlab script:

% Open directory

cd (uigetdir)

% Open filtered image of CFP channel

A=imread (uigetfile);

B= mat2gray (A, [0 255]);

imshow (B)

% Open filtered image of YFP channel

C=imread(uigetfile);

D= mat2gray (C, [0 255]);

figure, imshow (D)

% Ratio YFP/CFP calculation

Ratio=D./B;

% Threshold

[a,b,c]=find (B<0.05);

s=size(a);

for i=1:s

Ratio(a(i,1),b(i,1))=0;

end

% Show ratio image

figure, imshow(Ratio)

colormap (jet)

*Establishment of the minimal volume in which it is possible to reliably measure FRET ratio changes*

In Figure 1G, we determined the minimal volume in which it is possible to reliably measure FRET ratio changes. In an axon (Figure 1F), we measured FRET responses after WIN 100nM addition, using different ROIs that correspond to different volumes. For large and medium ROIs, to calculate the volume (V) corresponding to a ROI, the axon was considered as a cylinder; the diameter (d) and length (L) were measured and volume was calculated as: V=π x L x (d/2)^2^. For small ROIs (smaller that axon diameter), in order to calculate the volume, the number of pixels included in the ROI (n) was measured and volume was calculated as: V=n x a^3^, with a: the length of a pixel.

**Supplementary Figure 1: Data distribution and experimental variability.**

**A-B-C:** FRET responses of neurons expressing AKAR4-Kras are shown as scatter graphs. Data for somata **(A)** are the same as in **Figure 2C_2_**. Data for dendrites **(B)** are the same as in **Figure 2D_2_**. Data for axons **(C)** are the same as in **Figure 3A_2_**. These scatter graphs show that the data distribution is Gaussian (as shown by the normality test in GraphPad Prism) and it is not possible to distinguish two groups of neurons, based on their responses to WIN or AM.

**A_1_-B_1_-C_1_:** All presented data are pooled from at least two independent experiments. In order to show the inter-experiment variability, we represented the responses from independent experiments in somata **(A_1_**, same data as in **Figure 2C_2_)**, dendrites **(B_1_**, same data as in **Figure 2D_2_)** and axons **(C_1_**, same data as in **Figure 3A_2_)**. One experiment on four (WIN #4) showed no decrease after WIN 100nM addition in the somatodendritic compartment but showed a strong decrease in the axon.
