# Supplementary figures and images for: Polarized cellular patterns of endocannabinoid production and detection shape cannabinoid signaling in neurons
Source: Front Cell Neurosci. 2015 Jan 6;8:426. doi: 10.3389/fncel.2014.00426 (PMC4285097; doi:10.3389/fncel.2014.00426)

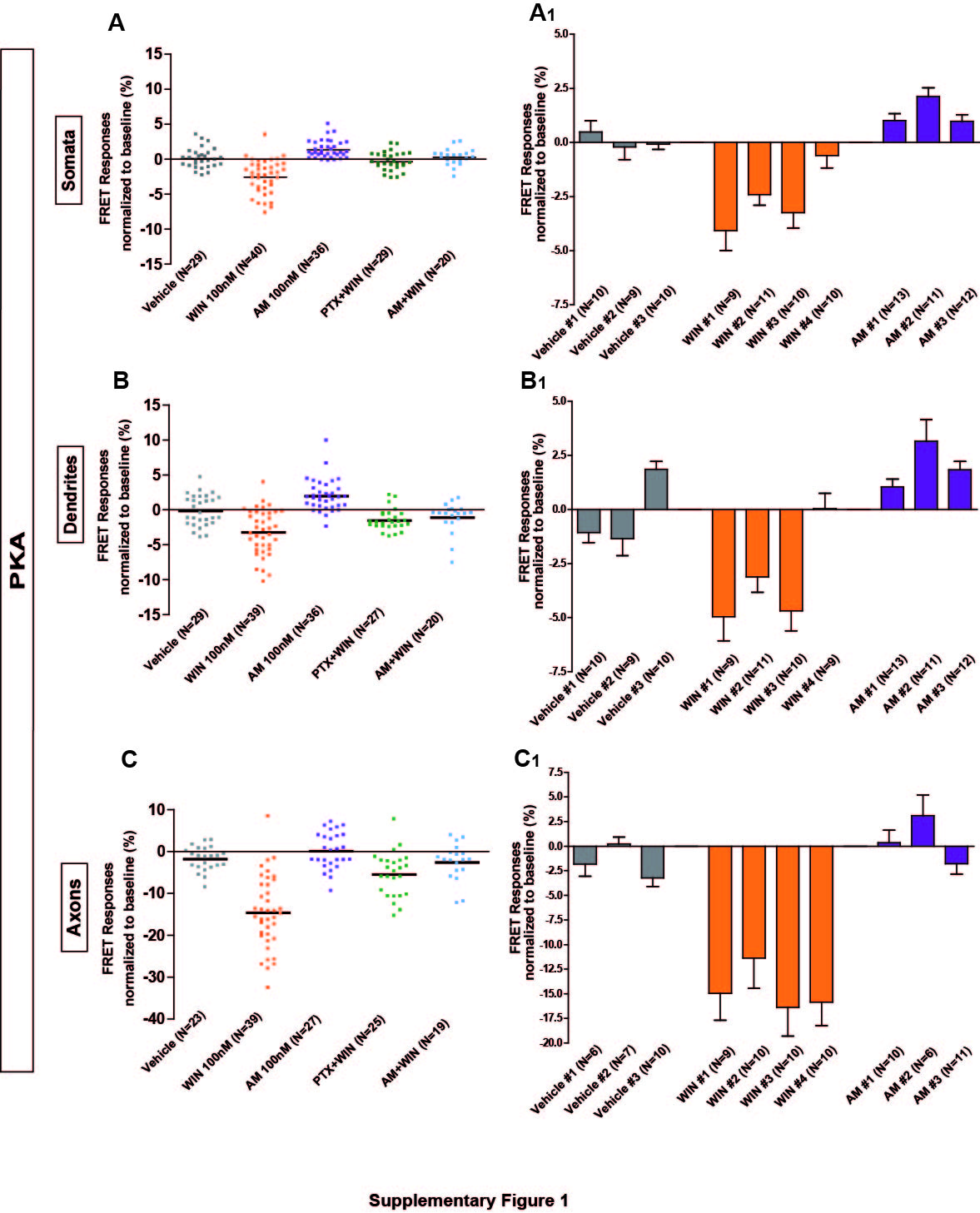

Supplement: Supplementary file 2 [file Image1.JPEG]
